# Revealing backward rescattering photoelectron interference of molecules in strong infrared laser fields

**DOI:** 10.1038/srep08519

**Published:** 2015-02-17

**Authors:** Min Li, Xufei Sun, Xiguo Xie, Yun Shao, Yongkai Deng, Chengyin Wu, Qihuang Gong, Yunquan Liu

**Affiliations:** 1 Department of Physics and State Key Laboratory for Mesoscopic Physics, Peking University, Beijing 100871, People's Republic of China; 2 Collaborative Innovation Center of Quantum Matter, Beijing 100871, China

## Abstract

Photoelectrons ionized from atoms and molecules in a strong laser field are either emitted directly or rescattered by the nucleus, both of which can serve as efficiently useful tools for molecular orbital imaging. We measure the photoelectron angular distributions of molecules (N_2_, O_2_ and CO_2_) ionized by infrared laser pulses (1320 nm, 0.2 ~ 1 × 10^14^ W/cm^2^) from multiphoton to tunneling regime and observe an enhancement of interference stripes in the tunneling regime. Using a semiclassical rescattering model with implementing the interference effect, we show that the enhancement arises from the sub-laser-cycle holographic interference of the contributions of the back-rescattering and the non-rescattering electron trajectory. It is shown that the low-energy backscattering photoelectron interference patterns have encoded the structural information of the molecular initial orbitals and attosecond time-resolved dynamics of photoelectron, opening new paths in high-resolution imaging of sub-Ångström and sub-femtosecond structural dynamics in molecules.

When exposing atoms and molecules in strong laser pulses, an electron wave packet can be tunneled through the Coulomb barrier suppressed by the laser fields^1^. The liberated electron wave packets may follow different paths from the ground state to the same final momentum, giving rise to the quantum interference effect. A firm understanding on the wave packet interference is pre-requisite for probing the electronic dynamic in atoms and molecules on the Ångström spatial and femtosecond-to-attosecond temporal scale^2^.

One of the most prominent interference effects in strong-field ionization is the interference of a repetitive wave-packet release at time intervals separated by the laser cycles. These intercycle interferences will lead to periodic peak structures in the electron energy spectrum, i.e., above-threshold ionization peaks^3^. Another type of interference that will contribute to the photoelectron angular distributions (PADs) of above threshold ionization is the intracycle interference of the electron wave packets released at adjacent half-cycles, which can either be ionized directly (direct trajectory) or turn around in the laser pulse (indirect trajectory)^4^, as illustrated in Fig. 1(a). The electron wave packets following the direct and indirect trajectories will have the same final momentum. The interference between the direct and indirect trajectories has been observed as a temporal double-slit experiment in a few-cycle laser pulse^5^,^6^.

The electrons after ionization can be driven back to the ion by the laser field and have an opportunity to rescatter with the nucleus^7^,^8^. The rescattering electron records detailed structural and dynamic information about the atoms and molecules, and thus it has been used to image the molecular orbital^9^,^10^ and to retrieve ultrafast dynamics of molecular structure^11^. The interference between the rescattering and non-rescattering trajectories are first revealed by the benchmark experiment performed for the metastable xenon atoms in deep tunneling regime^2^ (the Keldysh parameter^1^ 
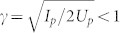
, *I_p_*: the ionization potential, *U_p_*: the ponderomotive potential, *U_p_ = F*_0_^2^/4*ω*^2^, *F*_0_: field amplitude; *ω*: field frequency, atomic units are used throughout otherwise specified.). The observed photoelectron holographic interference pattern reveals a characteristic spider-like structure, which originates from the interference of electron wave packets launched at the same quarter-cycle of the laser pulse with different transverse momenta (forward scattering photoelectron holography). Subsequently, the scaling of the holographic interference patterns depending on the laser duration, wavelength and intensity is reported^12^. It is shown that the holographic interference is favored by longer wavelengths and higher laser intensities^13^. In the multiphoton ionization regime (*γ* > 1), the interference of the side lobes observed in the PADs of above-threshold ionization have been revealed with the help of the quantum-trajectory Monte Carlo approach^14^.

Because of multiple centers and strong Coulomb focusing effects, the rescattering cross section in strong-field ionization of molecules is larger compared to atoms. Recently, Bian *et al*.^15^,^16^ predicted that the interference among the backward rescattering trajectories and the non-rescattering trajectories for strong-field ionization of molecules can be resolved for H_2_^+^ by numerically solving the time-dependent Schrödinger equation. In this paper, we present the experimental measurement of PADs of molecules (N_2_, O_2_ and CO_2_) at 1320 nm in the intensity region 0.2 ~ 1 × 10^14^ W/cm^2^. In the multiphoton regime, the ionization of molecules shows similar PADs with those of atoms. Increasing the laser intensity, we observe that the PADs of molecules reveal a novel pattern, i.e., interference stripes. Using a semiclassical rescattering model with including the interference effect, we show that the stripe pattern is related to the enhancement of the subcycle interference between the long rescattered trajectory and the indirect trajectory when going to the tunneling regime. The observation provides the first unambiguously experimental evidence of the backward rescattering on photoelectron angular distributions, enriching the photoelectron holography using strong laser fields. We further show that the low-energy backscattering photoelectron holography has encoded the structural information of the molecular initial orbitals and sub-femtosecond information of electronic dynamics.

## Results

Figure 2 shows the measured two-dimensional PADs in momentum space (*P_z_*, *P*_⊥_) of O_2_, N_2_, and CO_2_ molecules at the intensities of 0.2 ~ 1 × 10^14^ W/cm^2^ at 1320 nm (Details of the experiment can be found in Methods). The *P*_z_ and *P*_⊥_ (
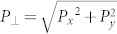
) represent the momentum distribution parallel and perpendicular to the laser polarization direction, respectively. At low laser intensities, i.e., in the multiphoton ionization regime, the PADs reveal ring-like multiphoton structures. The PADs of molecules reveal similar low-energy resonant structure at the wavelength of 800 nm^17^,^18^,^19^. At 1320 nm, the PADs of molecules (see the top panels of Fig. 2) are similar to those of atoms at low laser intensities^20^. Interesting, with increasing the laser intensity, an interference stripe structure is enhanced for strong-field ionization of molecules, which is nearly perpendicular to the laser polarization axis. The fringe spacing between adjacent interference stripes is almost 0.1 a.u., as shown by the white dashed lines in Fig. 2. This interference stripe pattern was not observed for the rare gas atoms^20^,^21^ and the negative molecular ions^22^ at similar laser intensities and wavelengths.

By projecting the two-dimensional PADs onto the laser polarization direction, one can clearly identify the enhanced interference structure in the *p_z_* momentum distributions with increasing the laser intensity, as shown in Fig. 3.

As mentioned above, the rescattering cross sections for molecules are larger than those of atoms and negative molecular ions. The possibility of backward rescattering will increase for molecules because of multiple centers and strong coulomb focusing effects^15^. The averaged scattering cross section for randomly aligned molecules is given by Ref. 23 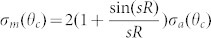, where *s* is the difference between the scattered and incident wave vectors, *R* is the internuclear distance of molecules, *θ_c_* is the scattering angle, and *σ_a_*(*θ_c_*) is the scattering cross section of atoms. According to the formula, the molecular scattering cross section is approximately twice as large as the atomic cross section for backward rescattering. Increasing the laser intensity, the holographic interference induced by the rescattering will become more important^13^. To resolve the underlying processes leading to the interesting interference pattern, we perform a simulation starting with the classical rescattering model^24^. We have further improved the model by including the interference effect^25^ and the weight of each trajectory according to the Ammosov-Delone-Krainov (ADK) tunneling theory^26^,^27^ (See Methods).

**Figure 1:**
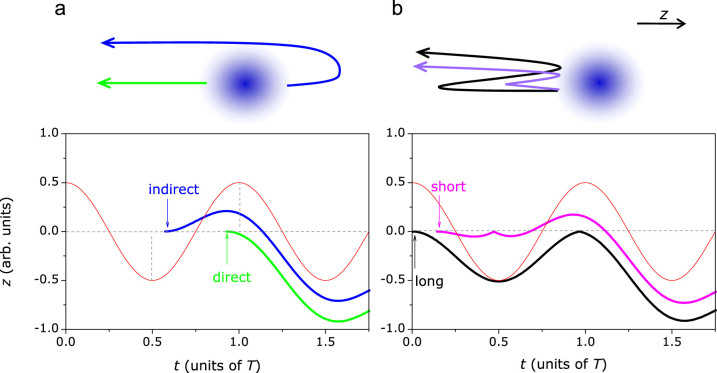
Illustration of the non-rescattering trajectories and the back-scattering trajectories. (a) There are two kinds of the non-rescattering trajectories in each laser cycle, i.e., the direct trajectory released before the field maximum (green) and the indirect trajectory released after the field maximum (blue). The indirect trajectory is initially emitted along the positive *z* direction and finally obtains a negative momentum. (b) According to the excurse time between the ionization and the rescattering, there are two types of backward rescattering trajectories, i.e., the long trajectory (black) and the short trajectory (magenta). In the bottom parts of (a) and (b), the arrows indicate the instant of the electron release. The scattering angle is assumed to be 180° in (b). The laser field is shown by the red curves in arbitrary units.

Similar to the high-order harmonic generation process, there are a long trajectory and a short trajectory with the same final momentum for the backward rescattering according to the excursion time between the ionization and the rescattering^25^,^28^, as illustrated in Fig. 1(b). Usually, the energy of the backward rescattering electrons are distributed in the energy range of 0 ~ 10*U*_p_. When the scattering angle is 180°, the electrons can obtain the maximum energy of 10*U_p_* at the tunneling phase of ~17° after the field maximum^8^. For other tunneling phases, the electrons can obtain smaller final energy. Only those rescattering trajectories with energy less than 2*U*_p_ can interfere with the non-rescattering trajectories. Both the long trajectory (released before 17°) and the short trajectory (released after 17°) can obtain final energy less than 2*U*_p_. The ionization probability of the long and the short trajectories depends on the laser phase at the instant of the tunnelling. Because the long trajectory is released near the field maximum, the ionization probability of the long rescattered trajectory will be much larger than that of the short rescattered trajectory for those lower-energy rescattering photoelectrons according to the ADK theory.

When the backscattered trajectories acquire the same final momentum as the non-rescattering trajectories, the interference will take place. Generally, there are four types of interferences between the backward rescattered and the non-rescattered trajectories, i.e., the long rescattered trajectories with the indirect trajectories, the long rescattered trajectories with the direct trajectories, the short rescattered trajectories with the indirect trajectories, and the short rescattered trajectories with the direct trajectories. We have calculated those four interference patterns for a model diatomic molecular ions with an internuclear distance of 2 a.u. for the perpendicular orientation at an intensity of 1 × 10^14^ W/cm^2^ (1320 nm), as shown in Fig. 4. In those plots, we do not include the trajectory weight in the calculation. We find that two interference patterns have comparable fringe spacing with the experiment, i.e., the interference between the long rescattered trajectory and the indirect trajectory [Fig. 4(a)], and the interference between the short rescattered trajectory and the direct trajectory [Fig. 4(d)]. The fringe spacing of the other two channels [Fig. 4(b) and 4(c)] is much larger.

The fringe spacing of the interference stripes is determined by the phase differences accumulated along the trajectories, which depend sensitively on the difference between the rescattering time of the rescattering trajectory and the birth time of the non-rescattering trajectory^25^. As shown in Fig. 1, because the time differences between the long rescattered trajectory and the indirect trajectory and between the short rescattered trajectory and the direct trajectory are considerably large, the fringe spacing for these two interferences is small and their interference patterns look quite similar. The much shorter time difference for the other two interference channels (the short rescattered trajectory with the indirect trajectory and the long rescattered trajectory with the direct trajectory) leads to much larger spacing of the fringes in *p_z_* momentum distributions.

Because there is no ionization probability for all of the trajectories in Fig. 4, the overall distribution of the calculated interference patterns looks like a curved structure. It is very necessary to include the ionization probability to study the relative contribution to the final PADs. Because the long rescattered trajectory are born near the field maximum, as seen in Fig. 1(b), the yield of the long rescattered trajectories is much higher than that of the short rescattered trajectories^25^. On the other hand, the yield of the indirect trajectories will dominate over that of the direct trajectories because of the effect of the long-range Coulomb potential^14^,^29^. Thus, the interference between the long rescattered trajectories and the indirect trajectories plays a dominant role in the formation of the low-energy interference stripes. In order to see the interference pattern more clearly, we assume that the pair of trajectories has the same weight and we further calculate the interference patterns for the long rescattered trajectory with the indirect trajectory, as shown in Fig. 5(a). The simulated interference stripes are nearly transverse to the laser polarization direction and the fringe spacing is almost 0.1 a.u., which is similar to the experimental data. The simulated interference patterns between the long rescattered trajectory and the indirect trajectory agree qualitatively with the interference stripes observed in the measured PADs.

## Discussions

**Figure 2:**
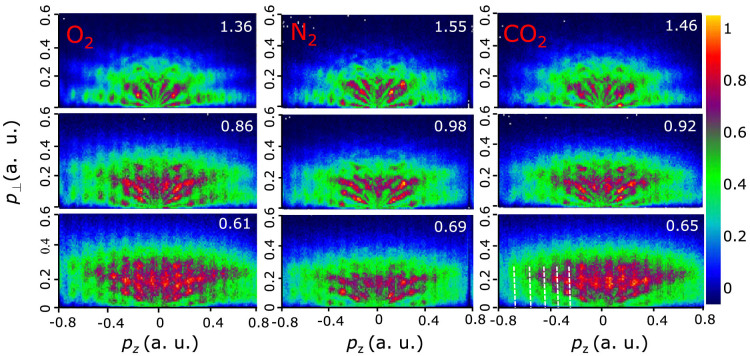
Experimental two-dimensional PADs. The measured two-dimensional PADs in momentum space (*p_z_*, *p*_⊥_) are shown for O_2_, N_2_ and CO_2_ in the intensities of 0.2–1 × 10^14^ W/cm^2^ at 1320 nm. The Keldysh parameters are labeled at the top right corner. The enhanced interference stripes are marked by the white dashed lines.

**Figure 3:**
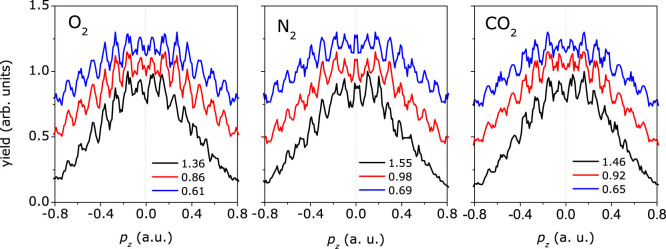
Experimental momentum distributions along the laser polarization direction. The measured *p_z_* momentum distributions of O_2_, N_2_ and CO_2_ are shown in the intensities of 0.2–1 × 10^14^ W/cm^2^ at 1320 nm. The Keldysh parameters are shown by the labels. The curves are separated slightly in the vertical direction for visual convenience. The gray dashed lines show the position *p_z_* = 0. One can find that it is an interference minimum for O_2_ and CO_2_, and a maximum for N_2_ when *p_z_* = 0.

**Figure 4:**
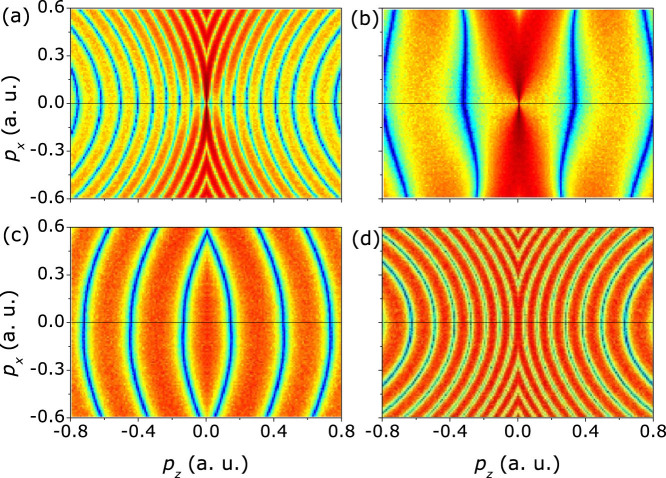
Simulated interference patterns without considering the ionization probability. The interference patterns of a two-center molecular ion between the long rescattered trajectory and the indirect trajectory (a), the long rescattered trajectory and the direct trajectory (b), the short rescattered trajectory and the indirect trajectory (c), and the short rescattered trajectory and the direct trajectory (d). In the simulation, there is no ionization probability for each trajectory. Top and bottom panels of each plot correspond to the photoelectron rescattered by the parent ions and the neighboring ions in the molecules, respectively. The internuclear distance is 2 a.u.. The intensity is 1 × 10^14^ W/cm^2^ and the wavelength is 1320 nm.

**Figure 5:**
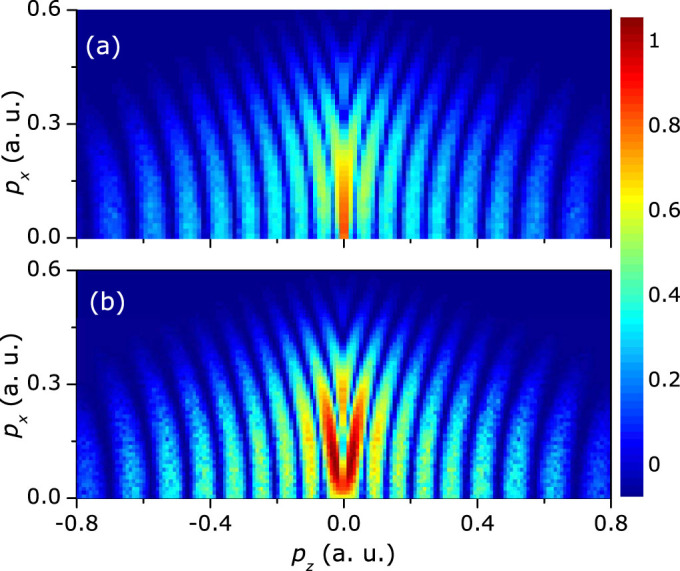
Simulated interference patterns between the long trajectory and the indirect trajectory. The additional phase difference is zero for σ_g_ orbital (a) and π for π_g_ orbital (b) (see text). The intensity is 1 × 10^14^ W/cm^2^ and the wavelength is 1320 nm.

It has been shown that the high-energy above-threshold ionization photoelectrons depend sensitively on the molecular outermost orbital^30^, which has been used to extract the electron-ion differential scattering cross section for molecules^31^. Different with the high-energy rescattering photoelectrons, the low-energy rescattering electrons are usually mixed with the non-scattering photoelectrons. We will show that the low-energy photoelectron rescattering holographic patterns is encoded with the molecular structure information, thus could also be used to probe the molecular structure. In Fig. 2, we find a striking interesting feature for different molecules at zero momentum of photoelectron angular distributions. It is an interference minimum for O_2_ and CO_2_, while it is an interference maximum for N_2_. This is more clearly seen in the momentum distribution projected onto the laser polarization direction, as shown in Fig. 3. The outermost orbitals of O_2_ and CO_2_ are the anti-bonding π_g_ geometry and that of N_2_ reveals the bonding σ_g_ geometry. There will be another phase difference of π for the tunneling from adjacent half cycles for the π_g_ initial orbital^32^, which will result in the destructive interference at the zero momentum, as shown in Fig. 5(b). The experimental results indicate that the interference of the long rescattered trajectories and the indirect trajectories is sensitive to the molecular structures. The low-energy rescattering photoelectron interference holography can also provide a useful probe of the molecular structure. Compared with the method of imaging molecular structure using the non-rescattering photoelectrons^10^, the backward rescattering photoelectron holography can survive in the randomly oriented molecules. In addition, each interference stripe corresponds to the birth time and the rescattering time of the photoelectrons in the semiclassical model. The low energy rescattering photoelectron holography has encoded the electron dynamics with subcycle resolution.

Previously, the imaging of the ultrafast structural dynamics in molecules (e.g., the changes of the bond length) is realized by tuning the laser wavelength, thus changing the excursion time between the ionization and the rescattering of photoelectrons^11^. In the backward rescattering holographic interference, the interference stripes have recorded both the molecular structure and the electron excursion time. Hence, it is possible to take ultrafast snapshot for the sub-Ångström structural changes in molecules by combining the information of the molecular structure with the photoelectron temporal information.

One should note that other interference channels will also have significant contributions to the PADs, e.g., the ring-like multiphoton ionization structure and the spider-like interference structure. These structures have been studied previously^2^,^3^,^21^. The backward rescattering interference pattern will superimpose on those characteristic structures. At lower laser intensities, the backward rescattering interference pattern will be buried below other more prominent interference patterns, e.g., the ring-like patterns. With increasing the intensity, the ring-like patterns will become blurred by the intensity averaging. As a result, the visibility of the backward rescattering photoelectron interference increases at high intensities for strong-field ionization of molecules.

The simulated backward rescattering interference pattern does not completely agree with the observed interference stripes, which may arise from the negligence of the long-range Coulomb potential after the electron ionization. The Coulomb tail has shown its importance for both the rescattering and non-rescattering photoelectrons^14^,^29^,^33^. Hence, the trajectories and the interference patterns will be inevitably modified by the Coulomb tail. Currently, the calculation with quantitatively including the Coulomb potential and the interference effect is considerably more complicated^2^,^12^,^14^, especially for the molecules.

In summary, we have measured the PADs of molecules in intense infrared laser fields. We experimentally observe the enhancement of a novel interference stripe pattern in strong-field ionization of molecules when going to the tunneling regime, which is associated to the interference between the back-rescattered and the non-rescattered trajectories because of the large rescattering cross section of molecular ions. Subcycle analysis indicates that the interference between the long rescattered trajectory and the indirect trajectory plays more important roles. It is well-known that imaging the dynamics of complex molecules with the help of the rescattering electron current, generated by tunneling ionization of the molecules, is one of intriguing interests in strong-field community. The low-energy rescattering interference was shown here as a potential tool for probing the molecular structures. Probably even more important, the interference stripe pattern encodes the sub-femtosecond electron and nuclear dynamics of the molecules, which offers the exciting opportunity to imaging the ultrafast structural dynamics in molecules with an unprecedented time resolution.

## Methods

### Experimental methods

A linearly polarized 1320 nm radiation was generated by an optical parametric amplification (OPA) system that was pumped by 25 fs, 795 nm pulses from a Ti:Sa laser system with 3 kHz repetition rate, amplified pulse energy up to 0.8 mJ. The estimated pulse duration at 1320 nm was ~30–35 fs. The two-dimensional PADs from single ionization of molecules are measured by a reaction microscope^20^ (for the principle see Ref. 34) with the photoelectron momentum resolution ~ 0.02 a.u. along the time-of-flight direction and ~0.05 a.u. along the transverse direction. Ions and electrons were measured with two position-sensitive microchannel plate detectors respectively. We applied weak electric (~3 V/cm) and magnetic (~5 G) fields along the time-of-flight axis to guide the electrons and ions to the detectors. The full momentum vectors of the particles were constructed by measuring the time of flight and the impact position on the detections. In the off-line analysis, the photoelectrons were selected in coincidence with their singly charged parent molecular ions to reduce the effect of background electrons. We have carefully adjusted the magnetic field and electrical field to avoid the poor resolution for the transverse momentum for those low-energy electrons. The laser polarization direction was along the time-of-flight axis. The molecular axis is randomly oriented with respect to the laser polarization direction.

### Theoretical methods

We assume that the laser pulse is cosine-like with **F**(*t*) = *F*_0_cos(*ωt*)**z**, where **z** is the laser polarization direction. The rescattering electrons are released in the laser phase (0, 17°) for the long rescattered trajectory and in the laser phase of (17°, 90°) for the short rescattered trajectory with zero initial momentum. The electron velocity before rescattering can be calculated as *v_z_*(*t*) = −*F*_0_/*ω*[sin(*ωt*) − sin(*ωt*_0_)]. The rescattering time *t_c_* is obtained by numerically solving the classical equation of motion^24^ cos(*ωt_c_*) − cos(*ωt*_0_^sig^) + *ω*(*t_c_* − *t*_0_^sig^)sin(*ωt*_0_^sig^) = 0. At *t*_c_, the electron elastically rescatters off the nucleus with a scattering angle with respect to its impact direction. The scattering angle *θ_c_* is randomly distributed within [0, 360°], which means that the rescattering electrons can be scattered in full solid angles at the instant of rescattering^25^. The asymptotic momentum of the rescattered electron is *p_z_* = *F*_0_/*ω*sin(*ωt_c_*) + *v_c_*cos(*θ_c_*) and, *p_x_* = *v_c_*sin(*θ_c_*), where *v_c_* = *v_z_*(*t_c_*) is the electron momentum at rescattering. For the non-rescattering photoelectrons, the birth time (*t*_0_^ref^) of the direct and indirect trajectories is expressed as [2*π* + sin^−1^(*ωp_z_*/*F*_0_)]/*ω* and [*π* − sin^−1^(*ωp_z_*/*F*_0_)]/*ω*, respectively. Those electrons will obtain a drift momentum *v_z_*(*t*) = −*F*_0_sin(*ωt*)/*ω*. The corresponding energy is smaller than 2*U*_p_. If the final drift momenta of the non-rescattering trajectories (direct and indirect trajectories) and the rescattering trajectories are the same, the interference will take place.

In the calculation, the phase of each trajectory is given by the classical action along the trajectory 
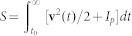
. The phase difference between the backscattered and the non-rescattered trajectories is 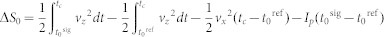. When the electron is rescattered by the neighboring core of the molecule, the pair of the trajectories will have an additional phase difference Δ*S*_1_ = *v_c_R*[cos(*β*) − cos(*β* + *θ_c_*)], where *β* is the angle between the molecular axis and the laser polarization direction (here we considered the perpendicular condition with *β* = 90°)^15^. In our model, each pair of trajectories is weighted by *W*(*t*_0_,*v*_0_) = *W*_0_(*t*_0_)*W*_1_(*v*_0_), in which 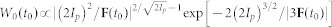 is the instantaneous tunnelling ionization probability, and 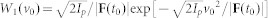 is the initial transverse momentum distributions^26^,^27^. We have sampled the electron ensemble with the Monte-Carlo method.

## Author Contributions

M.L., Y.D., C.W., Q.G. and Y.L. designed the experiment and carried out the measurement; M.L., X.S., X.X., Y.S. and Y.L. discussed the results; M.L. and Y.L. provided the theoretical parts and wrote the manuscript; all authors reviewed the manuscript.

## References

[b1] Keldysh L. V. Ionization in the field of a strong electromagnetic wave. Sov. Phys. JETP 20, 1307–1314 (1965).

[b2] Huismans Y. *et al.* Time-resolved holography with photoelectrons. Science 331, 61 (2011). 21163963 10.1126/science.1198450

[b3] Agostini P., Fabre F., Mainfray G., Petite G. & Rahman N. K. Free-free transitions following six-photon ionization of Xenon atoms. Phys. Rev. Lett. 42, 1127–1130 (1979).

[b4] Arbó D. G., Persson E. & Burgdörfer J. Time double-slit interferences in strong-field tunneling ionization. Phys. Rev. A 74, 063407 (2006).

[b5] Lindner F. *et al.* Attosecond double-slit experiment. Phys. Rev. Lett. 95, 040401 (2005). 16090782 10.1103/PhysRevLett.95.040401

[b6] Gopal R. *et al.* Three-dimensional momentum imaging of electron wave packet interference in few-cycle laser pulses. Phys. Rev. Lett. 103, 053001 (2009). 19792493 10.1103/PhysRevLett.103.053001

[b7] Schafer K. J., Yang B., DiMauro L. F. & Kulander K. C. Above threshold ionization beyond the high harmonic cutoff. Phys. Rev. Lett. 70, 1599–1602 (1993). 10053336 10.1103/PhysRevLett.70.1599

[b8] Corkum P. B. Plasma perspective on strong-field multiphoton ionization. Phys. Rev. Lett. 71, 1994–1997 (1993). 10054556 10.1103/PhysRevLett.71.1994

[b9] Itatani J. *et al.* Tomographic imaging of molecular orbitals. Nature 432, 867–871 (2004). 15602553 10.1038/nature03183

[b10] Meckel M. *et al.* Laser-induced electron tunneling and diffraction. Science 320, 1478 (2008). 18556555 10.1126/science.1157980

[b11] Blaga C. I. *et al.* Imaging ultrafast molecular dynamics with laser-induced electron diffration. Nature 483, 194–197 (2011). 22398558 10.1038/nature10820

[b12] Huismans Y. *et al.* Scaling laws for photoelectron holography in the midinfrared wavelength regime. Phys. Rev. Lett. 109, 013002 (2012). 23031101 10.1103/PhysRevLett.109.013002

[b13] Marchenko T., Huismans Y., Schafer K. J. & Vrakking M. J. J. Criteria for the observation of strong-field photoelectron holography. Phys. Rev. A 84, 053427 (2011).

[b14] Li M. *et al.* Classical-quantum correspondence for above threshold ionization. Phys. Rev. Lett. 112, 113002(2014). 24702359 10.1103/PhysRevLett.112.113002

[b15] Bian X. B. & Bandrauk A. D. Attosecond time-resolved imaging of molecular structure by photoelectron holography. Phys. Rev. Lett. 108, 263003 (2012). 23004974 10.1103/PhysRevLett.108.263003

[b16] Bian X. B. *et al.* Subcycle interference dynamics of time-resolved photoelectron holography with midinfrared laser pulses. Phys. Rev. A 84, 043420 (2011).

[b17] Rudenko A. *et al.* Resonant structures in the low-energy electron continuum for single ionization of atoms in the tunneling regime. J. Phys. B- At. Mol. Opt. Phys. 37, L407–L413 (2004).

[b18] Maharjan C. M., Alnaser A. S., Litvinyuk I., Ranitovic P. & Cocke C. L. Wavelength dependence of momentum-space images of low-energy electrons generated by short intense laser pulses at high intensities. J. Phys. B- At. Mol. Opt. Phys. 39, 1955–1964 (2006).

[b19] Deng Y. *et al.* Differential study on molecular suppressed ionization in intense linearly and circularly polarized laser fields. Phys. Rev. A 84, 065405 (2011).

[b20] Liu H. *et al.* Low yield of near-zero-momentum electrons and partial atomic stabilization in strong-field tunneling ionization. Phys. Rev. Lett. 109, 093001 (2012). 23002830 10.1103/PhysRevLett.109.093001

[b21] Hickestein D. D. *et al.* Direct visualization of laser-driven electron multiple scattering and tunneling distance in strong-field ionization. Phys. Rev. Lett. 109, 073004 (2012). 23006367 10.1103/PhysRevLett.109.073004

[b22] Hultgren H. & Kiyan I. Yu. Photodetachment dynamics of F_2_^−^ in a strong laser field. Phys. Rev. A 84, 015401 (2011).

[b23] Cornaggia C. Molecular rescattering signature in above-threshold ionization. Phys. Rev. A 78, 041401(R) (2008).

[b24] Paulus G. G., Becker W., Nicklich W. & Walther H. Rescattering effects in above-threshold ionization: a classical model. J. Phys. B- At. Mol. Opt. Phys. 27, L703–L708 (1994). 10.1103/physreva.52.4043 9912718

[b25] Li M. *et al.* Recollision-induced subcycle interference of molecules in strong laser fields. Phys. Rev. A 89, 033425 (2014).

[b26] Ammosov M. V., Delone N. B. & Krainov V. P. Tunnel ionization of complex atoms and of atomic ions in an alternating electromagnetic field. Sov. Phys. JETP 64, 1191–1194 (1986).

[b27] Delone N. B. & Krainov V. P. Energy and angular electron spectra for the tunnel ionization of atoms by strong low-frequency radiation. J. Opt. Soc. Am. B 8, 1207–1211 (1991).

[b28] Lewenstein M., Salières P. & L'Huillier A. Phase of the atomic polarization in high-order harmonic generation. Phys. Rev. A 52, 4747–4754 (1995). 9912816 10.1103/physreva.52.4747

[b29] Li M. *et al.* Subcycle dynamics of Coulomb asymmenty in strong elliptical laser fields. Phys. Rev. Lett. 111, 023006 (2013). 23889395 10.1103/PhysRevLett.111.023006

[b30] Okunishi M. *et al.* Two-source double slit interference in angle-resolved high-erengy above-threshold ionization specta of diatioms. Phys. Rev. Lett. 103, 043001 (2009). 19659347 10.1103/PhysRevLett.103.043001

[b31] Okunishi M. *et al.* Extraction electron-ion differential scattering cross sections for partially aligned molecules by laser-induced rescattering photoelectron spectroscopy. Phys. Rev. Lett. 106, 063001 (2011). 21405464 10.1103/PhysRevLett.106.063001

[b32] Müth-Bohm J., Becker A. & Faisal F. H. M. Suppressed molecular ionization for a class of diatomics in intense femtosecond laser fields. Phys. Rev. Lett. 85, 2280 (2000). 10977991 10.1103/PhysRevLett.85.2280

[b33] Yuan J., Li M., Sun X., Gong Q. & Liu Y. Tunneling coordinates of high-energy photoelectrons in above-threshold ionization. J. Phys. B- At. Mol. Opt. Phys. 47, 015003 (2014).

[b34] Ullrich J. *et al.* Recoil-ion and electron momentum spectroscopy: reaction-microscopes. Rep. Prog. Phys. 66, 1463–1545 (2003).

